# Isoeffect calculations with the linear quadratic and its extensions: An examination of model-dependent estimates at doses relevant to hypofractionation

**DOI:** 10.4103/0971-6203.79689

**Published:** 2011

**Authors:** Frederick W. McKenna, Salahuddin Ahmad

**Affiliations:** Department of Radiation Oncology, University of Oklahoma Health Sciences Center, Oklahoma City, OK, USA

**Keywords:** Dose fractionation, hypofractionation, linear quadratic

## Abstract

The linear quadratic is the standard model for calculating isoeffects in the range of conventional dose per fraction. However, the use of hypofractionation and stereotactic body radiation therapy can call for isoeffect calculations for large doses per fraction. The purpose of this work is to investigate the linear quadratic at large doses per fraction. The linear quadratic is compared to models that incorporate effects such as dose protraction, whose purpose is to extend the useful range of the linear quadratic to larger doses. The linear quadratic and extended linear quadratic models are fit to 4 data sets. The model-predicted isoeffects for these data sets are calculated. It is found that the linear quadratic and extended linear quadratic predict different isoeffect curves for certain data sets. However, for these data sets, by appropriate selection of a α/β ratio, the linear quadratic can well approximate the extended linear quadratic models. In particular, it is found that a α/β ratio of 0.5 well approximates the extended linear quadratic isoeffect curve for 2 prostate cell lines for conventional and moderate doses per fraction.

## Introduction

The use of hypofractionation and stereotactic body radiation therapy[1–3] brings new demands on our understanding of cell survival at high doses. One such demand is for cell-survival models that can span the range from conventional doses per fraction to large doses per fraction. The two-parameter linear quadratic [linear quadratic (LQ): ln S = -α d - β d^2^] is the preferred model for cell survival at doses conventionally used in radiation therapy,[4] but it has also been used to model cell survival at large doses. For what cells and for what irradiation conditions the LQ can be used at large doses is still a matter of investigation.[5–7]

It is known that some cell lines demonstrate linear or weakly nonlinear falloff at high doses rather than the quadratic falloff predicted by the LQ.[8] A possible contributing factor to this falloff is that at high doses partial repair during irradiation is possible. This effect is referred to as dose protraction. Dose protraction has been incorporated into the linear quadratic model, which allows for a non-quadratic falloff of survival at large doses.[9,10] This extends use of the linear quadratic to higher doses. A further extension of the linear quadratic has been made by Guerrero and Li[11] so that it agrees more closely with the lethal–potentially-lethal theory[12] of cell survival. These models while requiring more parameters than the LQ should be more useful over extended dose ranges.

One purpose of a survival model such as the LQ is to predict what hypofractionated schedules will give the same outcome as a conventional treatment schedule. For example, Liu *et al*.[13] have used the linear quadratic to calculate hypofractionated and stereotactic schedules. However, using the linear quadratic to predict isoeffects for those cells demonstrating non-quadratic falloff at high doses may lead to less-than-ideal treatment schedules. Using the extended dose range models may result in different isoeffect predictions than the LQ. Guerrero and Li,[11] for example, used their extended linear quadratic theory to analyze measured isoeffect data. The LQ and extended linear quadratic provided different fits and therefore made different isoeffect predictions for some data sets.

The purpose of this study is to examine the isoeffect predictions of the linear quadratic (LQ) model and an extended linear quadratic (ELQ) model. The isoeffect studied is a cell-killing isoeffect derived from fitting the LQ and ELQ to 4 sets of cell-survival data. Attention is given to what extent the LQ can be used for isoeffect calculations and when should more complex models be used.

## Material and Methods

### 

#### The linear quadratic and dose protraction

The linear quadratic with the dose protraction factor is[10]

(1). $$\mathrm{InS}\;=\;-\alpha d\;-\;\beta Gd^{2}$$

The dose protraction factor G is given by

(2), $$G=\frac{2}{d^{2}}\int_{-\infty }^{\infty }dt\int_{t}^{\infty }duR\left(t\right)e^{-\lambda \left(u-t\right)}R\left(u\right)$$

where λ is the characteristic repair rate and R(t) = d{d(t)}/dt is the dose rate. For a constant dose rate, R(t) = R; and G is

(3), $$G=\frac{2}{{\left(\lambda T\right)}^{2}}\left(e^{\lambda T}\;+\;\lambda T\;-1\right)$$

where T (=d/R) is the treatment time. Inserting equation 3 into equation 1 results in

(4), $$\mathrm{In}S\;=\;-\left[\alpha +2\beta \frac{R}{\lambda }\right]d\;+\;2\beta {\left[\frac{R}{\lambda }\right]}^{2}-\;2\beta {\left[\frac{R}{\lambda }\right]}^{2}e^{-\lambda d/R}$$

Formulas similar in structure to equation 4 but with somewhat different physical interpretation have been proposed. Haynes[14] wrote a formula similar in structure to equation 4 but for acute doses of radiation. Guerrero and Li[11] proposed a modification of equation 4 that incorporates both equation 4 and an acute dose formula similar to the one written by Haynes. To allow for the possibility for these alternative physical pictures, a model similar in form to equation 4 but with an empirical parameter set will be used. Similar to equation 4, a parameter α will correspond to the slope of the model at low doses, and a parameter β will be a low dose quadratic parameter. The third parameter will be the slope at high doses, which will be given by α + γ. Using these parameters, the model becomes

(5). $$\mathrm{In}S\;=\;-\left(\alpha +\gamma \right)d\;+\;\frac{1}{2}\frac{\gamma^{2}}{\beta }-\frac{1}{2}\frac{\gamma^{2}}{\beta }e^{-2\;\beta d/\gamma }$$

These parameters will be used in order to more easily relate the parameters to the behavior seen in the plots of the data. In the Guerrero and Li[11] theory, γ = 2 β/(δ + λ/ R), where δ is a dose-rate–independent parameter, which is zero in equation 4. Unlike equation 4, γ can be finite for an infinite dose rate.

For large γ and low doses, equation 5 becomes

(6). $$\mathrm{In}S\;\approx \;-\alpha d-\beta d^{2}+\frac{2}{3}\frac{\beta }{\gamma }d^{3}+\ldots$$

For small γ and high doses, equation 5 becomes

(7). $$\mathrm{In}S\;\approx \;-\left(\alpha +\gamma \right)d\;+\frac{1}{2}\frac{\gamma^{2}}{\beta }$$

At high doses, equation 5 demonstrates linear behavior with a slope of α + γ and an extrapolation number of γ^2^/2β.

In the limit of infinite γ, equation 5 reduces to the two-parameter form

(8). $$\mathrm{In}S\;=\;-\alpha d\;-\beta d^{2}$$

Equation 5 will be designated as ELQ, whereas equation 8 will be referred to as LQ. The ELQ predicts that log survival should be linear with high dose, whereas the LQ predicts that survival should be quadratic.

#### Cell lines and fitting

The cell-survival data investigated is as follows: (1) a Chinese hamster ovary line, CHO, which was measured by Bartkowiak *et al*.[15] (dose rate, 0.5 Gy/min); (2) a lung cell line NCIH841, which was measured by Carmichael *et al*.[16] (dose rate, 2 Gy/min); (3) a prostate cell line, PC3, which was measured by Deweese *et al*.[17] (dose rate, 1 Gy/min); and (4) a prostate cell line, CP3, which was measured by Garcia *et al*.[18](dose rate, 1.9 Gy/min). These data sets were fit with the LQ and ELQ models. The fitting procedure used is discussed by McKenna and Ahmad.[19] For purposes of fitting, if error bars are not readily available, it is assumed that the standard deviation of a point is proportional to the value of the point. The fits give the α and β parameters for the LQ; and the α, β and γ parameters for the ELQ. No constraints were placed on the parameters. This resulted in some of the α parameters in the ELQ fits being negative. If this occurred, the fit was repeated with the α parameter fixed to zero.

#### Calculation of isoeffects

An isoeffect is a set of fraction numbers (n) and total doses (D) that results in the same effect. The LQ isoeffects are found by solving[4]

(9) $$E\;=\;\alpha D+\beta \frac{1}{n}D^{2}$$

for the set D and n that gives the same effect E. In equations 9 and 12 below, complete repair of sublethal damage between fractions and no cell growth during treatment are assumed. An isoeffect investigated herein is that achieved by 40 fractions of 2 Gy. For this case, E is

(10). $$E\;=\;\alpha \left(40\times 2\right)+\beta \;\frac{1}{40}{\left(40\times 2\right)}^{2}$$

Solving equation 9 for D gives

(11). $$D\;=\;\frac{1}{2/\left(\alpha /\beta \right)}\left[\sqrt{n^{2}\;+\frac{4nE/\alpha }{\alpha /\beta }}-n\right]$$

Equation 11 gives the total dose as a function of n that gives the effect E.

For the ELQ, the isoeffects are found by solving

(12), $$\left(\alpha \;+\;\gamma \right)D\;-\;n\frac{\gamma^{2}}{2\beta }+n\frac{\gamma^{2}}{2\beta }e^{-2\beta D/n\gamma }\;=\;E$$

where α, β and γ are from the fits. For the effect of 40 fractions of 2 Gy, E is

(13). $$E\;=\;\left(\alpha \;+\;\gamma \right)80\;-\;40\frac{\gamma^{2}}{2\beta }+40\frac{\gamma^{2}}{2\beta }e^{-2\beta 80/40\gamma }$$

Equation 12 is an implicit equation and therefore must be solved by either numerical or graphical methods.

Equations 11 and 12 are used to find the total dose D given in n fractions for fractions from n = 1 to n = 50.

## Results

**Figure 1:**
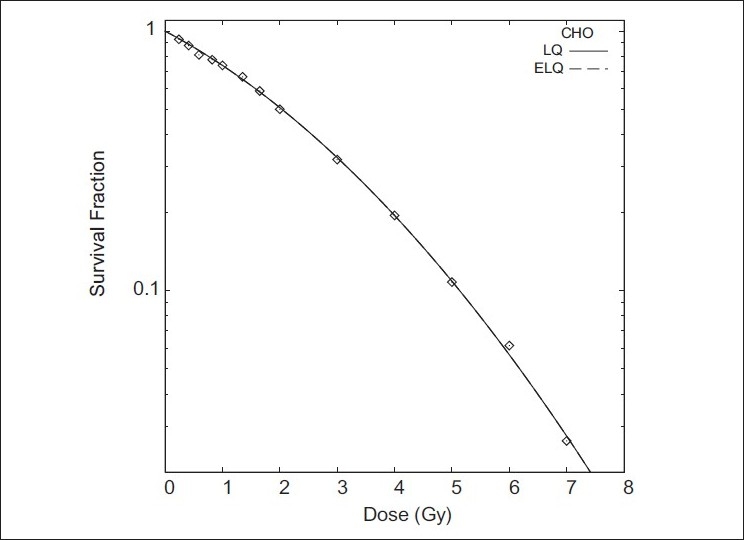
Fits of LQ and ELQ to CHO data. Parameters for fits are in Table 1. The ELQ reduces to the LQ fit by setting γ to infinity

**Figure 2:**
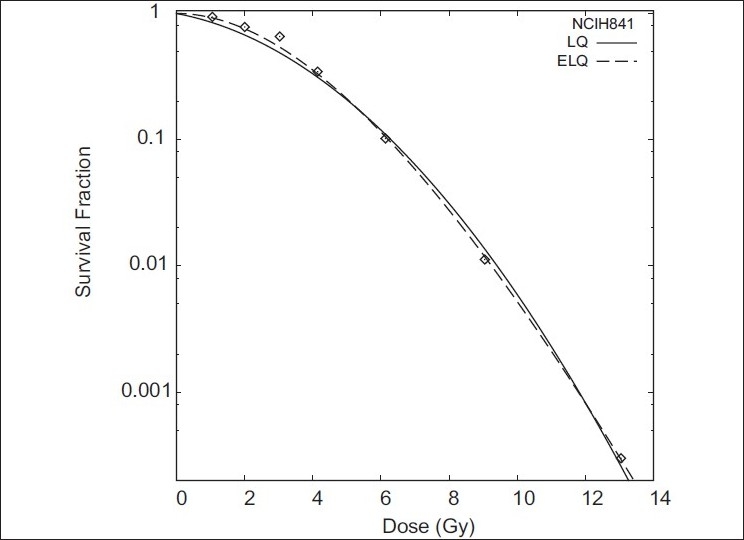
Fits of LQ and ELQ to NCIH841 data. Parameters for fits are in Table 1

**Figure 3:**
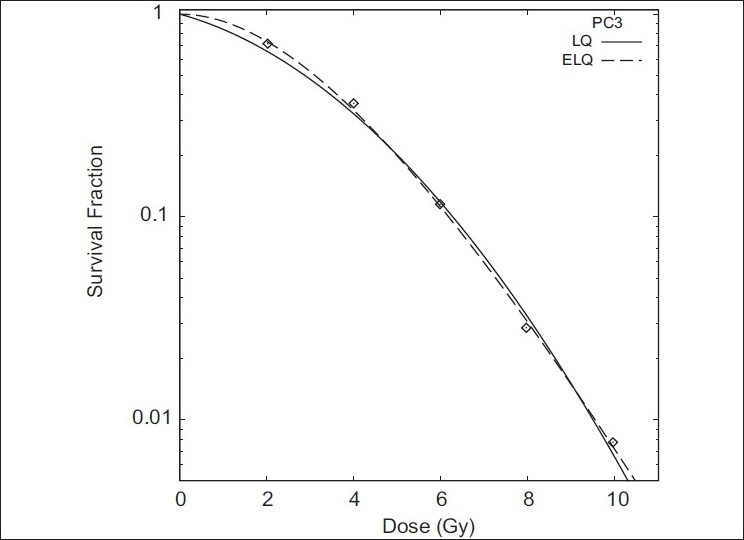
Fits of LQ and ELQ to PC3 data. Parameters for fits are in Table 1

**Figure 4:**
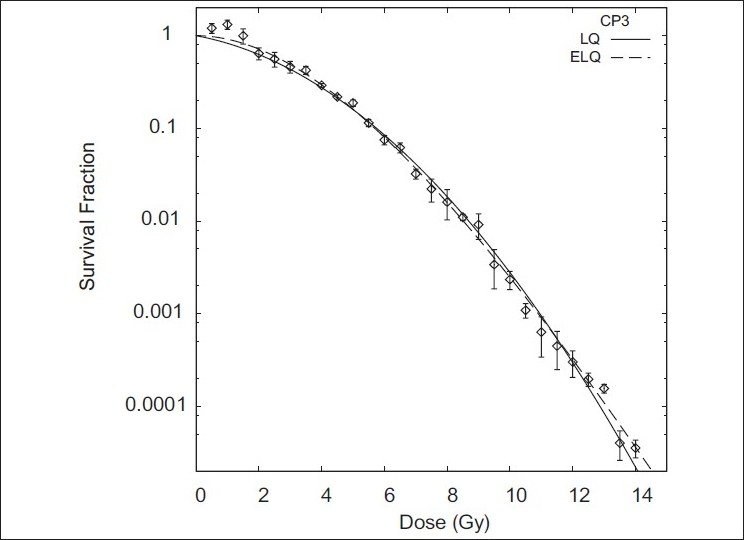
Fits of LQ and ELQ to CP3 data. Parameters for fits are in Table 1

**Figure 5:**
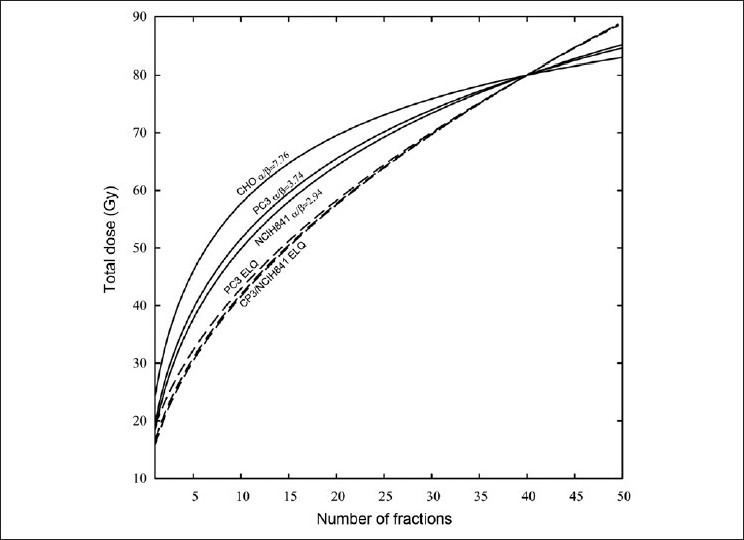
Isoeffect curves that give a predicted effect equivalent to 40 fractions of 2 Gy. Solid lines are isoeffects according to the LQ and dashed lines are isoeffects according to the ELQ model. The lines are labeled with the cell and model used to calculate them. The CP3 and NCIH841 ELQ lines almost overlap and so are labelled together

Figures 1–4 show the LQ and ELQ fits to the CHO, NCIH841, PC3 and CP3 data, respectively, and Table 1 gives the parameter values for the fits. In Table 1, WSSR stands for weighted sum of squared residuals and is a measure of the goodness of fit. The number in parenthesis next to the parameter value is the uncertainty in the parameter value. For NCIH841, PC3 and CP3, the ELQ fits give a smaller WSSR than the LQ fits. The ELQ WSSR for PC3, for example, is about 27% of the LQ WSSR. Whether a smaller WSSR is relevant depends on whether or not the two models provide different predictions. If model predictions are equivalent, one would generally use the simpler model. The LQ WSSR and ELQ WSSR are basically equal for the CHO fits. As is seen in Figure 1, the ELQ fit to the CHO data reduces to the LQ fit; this corresponds to the γ value being infinite in Table 1. For the NCIH841, PC3 and CP3 data, the α parameters for the ELQ fits were negative. So parameters were fixed to zero, as discussed in the ‘Material and methods’ section. Based on the parameters in Table 1, model-dependent isoeffects equivalent to 40 fractions of 2 Gy were calculated using equation 11 for the LQ fits and equation 12 for the ELQ fits. Table 2 gives selected values for these calculations. The calculations are also shown graphically in Figure 5.

**Table 1:** Summary of results for fitting the LQ and ELQ models to the data sets. The numbers in parenthesis are the percentage uncertainty in the parameter values.

	*LQ*			*ELQ*			
	*WSSR^1^*	α*(Gy^-1^)*	β*(Gy^-2^)*	*WSSR1*	α*(Gy^-1^)*	β*(Gy^-2^)*	γ*(Gy^-1^)*
CHO	0.011	0.2697 (3.35)	0.03476 (4.57)	0.011	0.269 (3.58)	0.0349 (4.6)	∞
NCIH841	0.174	0.1164 (30.6)	0.0396 (8.75)	0.038	0	0.07477 (5.33)	1.282 (9.6)
PC3	0.062	0.1371 (30.9)	0.03663 (14.1)	0.017	0	0.0885 (8.5)	0.856 (10.8)
CP3	67.0	0.1454 (11.4)	0.0443 (4.32)	36.4	0	0.0911 (3.33)	1.298 (5.47)

1 WSSR is the weighted sum of squared residuals

**Table 2:** Isoeffect calculations for selected fraction numbers for the LQ and ELQ models

*fx*	*CHO*	*NCIH841*		*PC3*		*CP3*		*LQ*	*LQ*
			
	*LQ*	*LQ*	*ELQ*	*LQ*	*ELQ*	*LQ*	*ELQ*	*α/β =1.0*	*α/β =0.5*
	*α/β =7.76*	*α/β =2.9*		*α/β =3.7*		*α/β =3.3*			
1	24.3	18.5	15.9	19.6	19.2	19.0	16.7	15.0	13.9
2	32.5	25.3	20.6	26.8	23.3	26.0	21.3	21.0	19.5
4	42.5	34.3	27.6	36.0	29.6	35.1	28.1	29.1	27.3
8	53.9	45.7	37.6	47.5	39.1	46.5	38.0	40.0	38.0
16	65.8	59.4	51.8	60.9	52.7	60.1	52.0	54.5	52.7
24	72.4	68.3	62.7	69.3	63.2	68.7	62.8	64.8	63.5
30	75.9	73.4	69.7	74.0	70.0	73.6	69.8	71.2	70.3
40	80	80	80	80	80	80	80	80	80
50	82.9	85.1	89.1	84.5	88.8	84.9	89.0	87.4	88.3

(The left-most column is the number of fractions. The numbers in the other columns represent the total dose in Gy that achieves the same effect as 40 fractions of 2 Gy. Also included in the table are isoeffect calculations for a/b = 0.5 and 1.0.)

## Discussion

For the CHO data, the ELQ reduces to the LQ. The CHO isoeffect curve shown in Figure 5 applies to both the LQ and ELQ models. For NCIH841, PC3 and CP3, the LQ- and ELQ-predicted isoeffect curves in Figure 5 differ. Since the chosen effect is that achieved by 40 fractions of 2 Gy, all curves must intersect at this point, as indeed they do. According to the LQ and ELQ models, other points on the curve will achieve the same effect as that of 40 fractions of 2 Gy.

**Figure 6:**
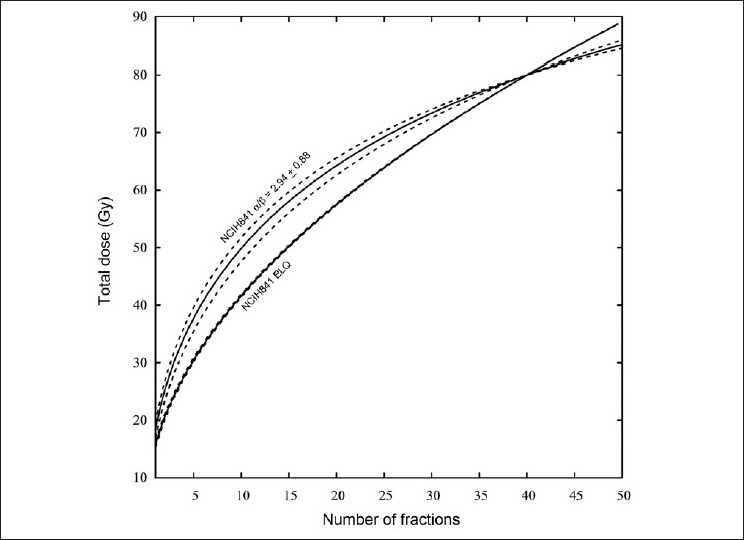
Uncertainty in the isoeffect curves based on the uncertainty in the parameters in Table 1. The parameters used are for the NCIH841 data. The isoeffect curves based on the LQ fit and the ELQ fit are shown. The uncertainty in the NCIH841 ELQ isoeffect curve is roughly the thickness of the line

The uncertainty in the parameters given in Table 1 means that there is an uncertainty in the isoeffect dose calculated with the model. As an example, the NCIH841 α is 0.1164 (30.6%), and β is 0.0396 (8.75%). The α/β with uncertainty is 2.94 (30%). Figure 6 shows the range of isoeffects based on the uncertainty in the parameters. The NCIH841 ELQ isoeffect is also shown. The uncertainty in the NCIH841 ELQ is about the thickness of the line. The NCIH841 LQ uncertainty is much greater than the NCIH841 ELQ uncertainty consistent with the larger uncertainty in the LQ parameters. Over a large range of fraction numbers, there is no overlap between the two curves.

In Figure 2, it is seen that at low doses the LQ curve is below the ELQ curve for NCIH841. One may think that this difference is small and so there should not be any practical difference between the prediction of LQ and ELQ models. However, Figure 5 and Table 2 show that this is not the case and that small differences at low doses can lead to large differences in predicting equivalent hypofractionated schedules. In Figure 5, the NCIH841 ELQ curve and the NCIH841 α / β=2.94 curve cross at the point D = 80 Gy and n = 40. Moving to larger or smaller fraction numbers, the two curves diverge. If, for example, a treatment schedule calling for 24 fractions is used (see Table 2), the LQ estimates that a total dose of 68.3 Gy is needed. On the other hand, the ELQ estimates that a total dose of 62.7 Gy is needed. This difference corresponds to about 2 fractions. An even more pronounced difference is seen if fewer fractions are used. Similar comments also apply to the PC3 and CP3 data.

### 

#### Model behavior at low doses

The reason for the difference between the isoeffect curves despite the similarities seen in Figures 2–4 can be traced back to the model predictions for cell survival at low doses. Table 3 shows the model predictions for the cell survival at 2 Gy. Compared to the LQ, ELQ predicts a higher cell survival. For the ELQ to achieve the same predicted effect as LQ, it is needed that

**Table 3:** Comparison of model predictions for the survival at 2 Gy

	*S_LQ_ (2 Gy)*	*S_ELQ_ (2 Gy)*	*^n^ELQ/ ^n^LQ*
NCIH841	0.676	0.758	1.41
PC3	0.657	0.734	1.36
CP3	0.626	0.717	1.41

(14), $$S_{ELQ}{\left(2Gy\right)}^{n_{ELQ}}\;=\;S_{LQ}{\left(2Gy\right)}^{n_{LQ}}$$

where n_ELQ_is the number of fractions needed to achieve the same predicted effect as that of n_LQ_. This ratio is shown in Table 3. For the 3 cells, approximately 1.4 times as many fractions are needed according to the ELQ model to achieve the same effect as that predicted by the LQ model. So, even the seemingly small difference in cell survival seen at low doses in [Figures 2–4] has a large effect on fraction numbers. This results in the different isoeffect curves seen in Figure 5 when 40 fractions of 2 Gy are used as the basis of comparison. This drop in S_LQ_ (2 Gy) compared to S_ELQ_ (2 Gy) is because the LQ is used to fit the high dose data. At these high doses, the survival does not decrease as rapidly as the quadratic term in the linear quadratic. In order to accommodate this slower-than-quadratic falloff, the α parameter is increased, causing a smaller value of S_LQ_ (2 Gy) than would occur if the LQ was only used for fitting low doses. Increasing the α parameter fits the large dose points better but sacrifices the fit at low doses.

#### The LQ limited to low doses

**Figure 7:**
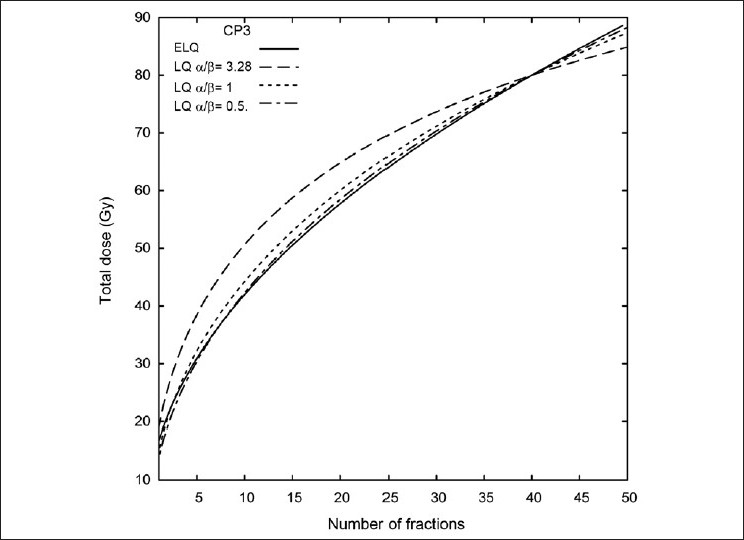
The CP3 ELQ model isoeffect curve compared to LQ isoeffect lines with small values of alpha / beta. A alpha / beta ratio of 0.5 approximates the ELQ curve over a range of fraction numbers

If the LQ is limited to only fitting the low dose data of NCIH841, PC3 and CP3, the resulting α / β ratio will be smaller than the ratios in Table 1. This will change the isoeffect curve. Figure 7 shows the isoeffect curve for the CP3 data as determined by the ELQ, by the CP3 α / β ratio from Table 1 (α / β = 3.28) and by some smaller α / β ratios. It is seen that as the α / β ratio gets smaller, the better it approximates the ELQ isoeffect curve. The prediction with α / β = 0.5 agrees well with the ELQ to within 1 Gy over the range of most of the plot. It is only at small fraction numbers that the prediction with α / β =0.5 dips below the ELQ curve and hence substantial disagreement occurs (> 1 Gy). A value of α / β for LQ can be found that will agree well with the ELQ isoeffect curve only over some range of fraction numbers. This useful α / β ratio will not necessarily be that found by fitting the LQ to an extended dose range data set. The range of fraction numbers over which the useful α / β agrees well with the ELQ depends also on the isoeffect itself as different isoeffects will need different α / β over different ranges.

**Figure 8:**
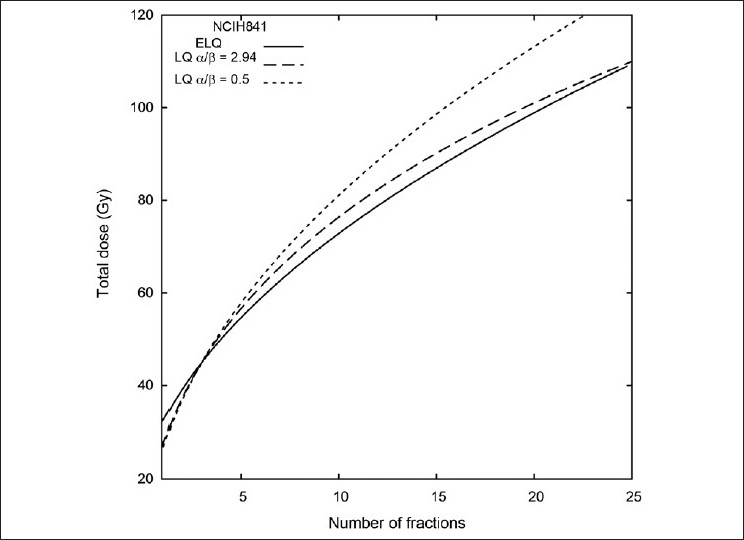
NCIH841 isoeffect curves that give a predicted effect equivalent to 3 fractions of 15 Gy. A small alpha / beta ratio (~0.5) no longer approximates the ELQ when large doses per fraction are used as the basis of the isoeffect calculations

To illustrate what can happen if very large doses per fraction are used, consider a schedule that calls for 3 fractions of 15 Gy, which has been used for the treatment of lung cancer.[20] According to the NCIH841 ELQ parameters in Table 1, 3 fractions of 15 Gy is equivalent to 72.8 Gy in 10 fractions and 98.9 Gy in 20 fractions. According to the LQ α / β =0.5(1.0), 3 fractions of 15 Gy is equivalent to 81(80) Gy in 10 fractions and 113(110) Gy in 20 fractions. On the other hand, using LQ α / β =2.94, which was found by fitting the LQ to the NCIH841 data, 3 fractions of 15 Gy is equivalent to 76.3 Gy in 10 fractions and 101 Gy in 20 fractions. These calculations are shown graphically in Figure 8. The larger LQ [α / β =2.94] approximates the ELQ better than the smaller values of α / β when considering large doses per fraction.

#### Prostate hypofractionation

Hypofractionation for the prostate radiotherapy is a topic of active investigation.[21,22] The questions involve how to calculate isoeffects for the prostate tumor and how to calculate isoeffects for the surrounding normal tissue. Brenner and Hall[23] and Fowler *et al*.[24] deduced a value of 1.5 for α / β for prostate tumors. Using assumptions different from those used by Brenner and Hall, Wang *et al*.[25] proposed a value of 3.1. Fowler[22] has suggested that α / β may be as low as 1. It was seen in the previous section that α / β = 0.5 is a good approximation for the ELQ CP3 prostate cell data for conventional-to-moderate doses per fraction. LQ fits to the extended dose range data resulted in α / β ratios in the 3-4 range. Compared to the ELQ isoeffects, α / β ratios in the 3-4 range called for substantially more dose compared to that arrived at by the ELQ calculations. While low LQ α / β ratios (.5-1) agreed with ELQ calculations for a range of fraction numbers, as discussed above, outside this range of fraction numbers ELQ calculations and the LQ calculations can disagree.

#### An alternative fit to PC3

The fitting performed here with the ELQ suggests that PC3 has a very small α / β ratio (practically 0). Carlson *et al*.[26] also analyzed the PC3 data, with different results. According to the radiobiological theory associated with equation 1, at very low dose rates, survival should be determined by S = exp (-α d). Besides the high dose rate data examined here, Deweese *et al*.[17] also measured survival data at low dose rates. Carlson *et al*. analyzed the PC3 data based on the requirement that equation 1 give a consistent parameter set for the high dose rate and low dose rate data. The parameters found by Carlson *et al*.[26] are α =.145 Gy^-1^, α / β = 4.11 and λ =.105/h. The survival curve with the parameters found by Carlson *et al*. closely resembles the LQ curve seen in Figure 3. Therefore, the Carlson *et al*. parameters will result in isoeffect calculations similar to those for the PC3 LQ (α / β =3.74) in Table 2. The question arises as to which parameter set gives more reliable isoeffect predictions. The Carlson *et al*. parameter set is consistent with the low and high dose rate data. This argues in favor of the LQ-type isoeffect curve seen in Figure 5. However, the ELQ parameter set in Table 1 gives a better fit to the high dose rate PC3 data. The ELQ parameter set found here might be dismissed as being due to a random fluctuation in a couple of data points. However, the basic pattern seen in the ELQ fit to the PC3 data is also seen in the NCIH841 and CP3 data, i.e., a very small (practically 0) value of α / β. In addition to the very small ratios seen here, the possibility of very small values of α / β has been noted by Guerrero and Li[11] in their analysis of measured isoeffect data using a formula similar to equation 5. As the ELQ with very small values of α / β gives different isoeffect calculations than the LQ with moderate α / β ratios (3-4), it is important to identify those situations with very small α / β.

## Conclusions

An examination of 4 data sets has been made with the LQ model and an extension of the LQ model, denoted ELQ. The CHO data set is representative of cell curves with moderate-to-large α / β ratios. It was well fit by the LQ, with no benefit coming from using the ELQ model. NCIH841, PC3 and CP3 are data sets which are representative of cell curves with low α / β ratios. The LQ and ELQ supply different fits and make different isoeffect predictions. The LQ α / β ratios for the fits to the NCIH841, PC3 and CP3 are in the range 3-4. However, this α / β may be too high since when the LQ is used to fit extended dose range data, the α parameter is increased and β parameter decreased to better fit the data at high doses. This sacrifices the fit at low doses. Rather, using α / β ratios in the range .5-1 results in LQ isoeffect predictions that approximate those of the ELQ for the NCIH841, PC3 and CP3 data. However, the low α / β LQ approximation to ELQ only holds for conventional and moderate doses per fraction. It may be a poor approximation to ELQ for large doses per fraction.

The isoeffects calculated herein are based on models for cell survival. The predicted isoeffect can depend on the model used. The models only estimate the isoeffect based on the information contained in the cell-survival data sets. The cell-survival data used here is based on *in vitro* cell-survival measurements. Preferably, the data would be based on *in vivo* cell-survival measurements. Preferable still would be actual clinical measurements of isoeffects at hypofractionated doses. Clinical isoeffects may differ from LQ estimates or ELQ estimates or both. Any model-based estimate must be compared against clinical outcomes.
